# Muscle and Liver Insulin Resistance Are Associated with Distinct Plasma Protein Profiles: Data from the DiOGenes Trial

**DOI:** 10.1210/clinem/dgaf562

**Published:** 2025-10-10

**Authors:** Esther J Kemper, Ellen E Blaak, Michiel E Adriaens, Ruth C R Meex

**Affiliations:** Department of Human Biology, NUTRIM Institute of Nutrition and Translational Research in Metabolism, Maastricht University Medical Centre+, 6200 MD Maastricht, The Netherlands; Department of Human Biology, NUTRIM Institute of Nutrition and Translational Research in Metabolism, Maastricht University Medical Centre+, 6200 MD Maastricht, The Netherlands; Maastricht Centre for Systems Biology, Maastricht University, 6211 LH Maastricht, The Netherlands; Department of Human Biology, NUTRIM Institute of Nutrition and Translational Research in Metabolism, Maastricht University Medical Centre+, 6200 MD Maastricht, The Netherlands

**Keywords:** obesity, tissue-specific insulin resistance, skeletal muscle, liver, proteomics, pathway enrichment analysis

## Abstract

**Context:**

Insulin resistance (IR) can develop in multiple organs, representing distinct etiologies toward cardiometabolic disease.

**Objective:**

This study aimed to investigate which proteins and pathways are specific for liver IR or muscle IR in individuals with overweight or obesity of the Diet, Obesity, and Genes cohort (n = 535) (NCT00390637, ClinicalTrials.gov).

**Methods:**

First, independent associations between muscle and liver IR and protein abundance levels were assessed. In the analysis, we corrected for study center, sex, body mass index, and age, whereby the analyses on liver IR were adjusted for muscle IR and vice versa. Differentially abundant proteins were then subjected to pathway enrichment analysis.

**Results:**

Muscle IR was associated with 160 proteins, and liver IR was associated with 81 proteins. Of these, 12 were shared between both forms of IR. Pathway enrichment analysis identified 51 enriched pathways for muscle IR, characterized by a strong inflammatory profile, including chemokine signaling, interleukin-6 signaling, and complement system-related pathways. In contrast, liver IR was associated with 11 enriched pathways, primarily related to the complement system.

**Conclusion:**

Understanding the processes underlying these distinct IR phenotypes could inform the development of personalized prevention strategies.

The global prevalence of individuals with overweight and obesity is increasing rapidly. In 2022, the World Health Organization estimated that 2.5 billon adults had a body mass index (BMI) of 25 kg/m^2^ or higher, classifying them as living with overweight, with 890 million of those individuals living with obesity (BMI ≥ 30 kg/m^2^) (1). Obesity is closely linked to chronic health complications, such as insulin resistance (IR) and type 2 diabetes (T2D), hepatic steatosis, cardiovascular diseases, and some types of cancer (2, 3). These health problems can lead to a reduced quality of life, increased health care costs, and premature death, and this highlights the importance of a better understanding of the mechanisms underlying these obesity-related health complications (4). While lifestyle interventions can be effective in preventing T2D and reducing cardiometabolic risk, the long-term outcomes are often limited, underscoring the need for additional preventive and targeted strategies.

IR, a hallmark of T2D, refers to the body's diminished response to the effects of insulin on glucose, lipid, and overall metabolism. Over the past decade, research has shown that IR can manifest systemically across all tissues or selectively in specific tissues, such as skeletal muscle and the liver. This results in some individuals having more pronounced muscle IR, while others primarily experience liver IR. These discordant phenotypes, which can be observed in individuals with overweight, even in the absence of fasting or postprandial hyperglycemia (5), may reflect different etiologies leading to T2D and cardiometabolic diseases and may benefit from tissue-specific treatment strategies (6). In this respect, previous research from our laboratory has shown that individuals with more pronounced liver IR have distinct metabolic characteristics compared to those with more pronounced muscle IR, specifically in their circulating metabolome profiles (6), lipidome (7), and subcutaneous adipose tissue transcriptome profiles (8). It has been found that muscle IR is primarily characterized by an enrichment of inflammatory pathways in subcutaneous adipose tissue and by enhanced systemic inflammation compared to liver IR (8). Additionally, it has been shown that these phenotypes respond differently to dietary macronutrient modulation in terms of insulin sensitivity and cardiometabolic health outcomes (9).

It is well known that various organs secrete products, including proteins, into the circulation. These proteins, such as hepatokines, myokines, and adipokines, influence metabolism in other organs through interorgan crosstalk (10-12). Previous research has demonstrated that the plasma proteome profile of individuals with IR differs from those with normal glycemic control (13). This raises the question whether the plasma proteome profile also varies depending on the type of tissue-specific IR.

Hence, in this study we aimed to investigate which proteins are independently associated with muscle IR or liver IR and the pathways these proteins are involved in. To achieve this, we determined the degree of liver IR and muscle IR in 535 individuals with overweight and obesity from the Diet, Obesity, and Genes (DiOGenes) study (NCT00390637, ClinicalTrials.gov) and performed proteomic analysis on their plasma to assess the independent associations of muscle and liver IR with protein abundance levels. Understanding these differences could provide further insights into the differential tissue-specific mechanisms of muscle and liver IR and support the development of targeted therapeutic strategies.

## Materials and Methods

### Study Design

Baseline data are used from the DiOGenes study, a multicenter, randomized, controlled dietary intervention study that involved 8 European countries. In total, 938 adults with overweight or obesity were recruited and completed the baseline examination between February 2006 and December 2007. Participants were required to meet the following inclusion criteria: age between 18 and 65 years, a BMI > 25 kg/m^2^, and fasting blood glucose concentrations < 6.1 mmol/L. Exclusion criteria included T2D and cardiovascular diseases. Specific details on recruitment, inclusion and exclusion criteria, and design and study procedures can be found elsewhere (14). All subjects gave written informed consent for participation in this study, and all experimental protocols were approved by local medical ethical committees (ClinicalTrials.gov: NCT00390637). All procedures were carried out according to the Declaration of Helsinki. The analyses described here include baseline data of 535 participants for whom plasma proteomics data and information on tissue-specific IR were available.

### Estimates of Tissue-specific IR

To estimate tissue-specific IR, participants underwent a standard 5-point oral glucose tolerance test (OGTT), and the muscle insulin sensitivity index (MISI) and hepatic IR index (HIRI) were calculated according to the method proposed by Abdul-Ghani et al, with minor modifications (15). Briefly, after an overnight fast, participants ingested 75 g glucose dissolved in 250 mL of water within a 5-minute period, after which blood samples were taken at 0, 30, 60, 90, and 120 minutes to determine glucose and insulin concentrations. The magnitude of the rise in blood glucose and insulin concentrations in the first 30 minutes following glucose ingestion is a reflection of the ability of insulin to suppress hepatic endogenous glucose production (15). Therefore, the HIRI was calculated as the product of the areas under the curve (AUCs) for glucose and insulin during this period; specifically, √(glucose0-30[AUC in mol/L· h] × insulin0-30[AUC in pmol/L· h]). The decline in blood glucose concentration after 60 minutes primarily reflects glucose uptake by peripheral tissues, mainly skeletal muscle (15). Therefore, the MISI was calculated as the rate of decline of the glucose concentration divided by the mean insulin concentration during the OGTT. The rate of decline was calculated as the slope of the least square fit to the decline in glucose concentration from peak to nadir (15). To determine MISI values, we utilized the MISI calculator, which applies the cubic spline method (16). This approach enhances the original method developed by Abdul-Ghani et al, as it uses a third-order polynomial for a more accurate fit of the glucose and insulin curves, thus providing a better estimation of the peak values. This method has been validated against the hyperinsulinemic-euglycemic clamp method (15, 16). After obtaining the MISI and HIRI indices, these values were scaled between 0 and 1, whereby 0 represents high insulin sensitivity and 1 represents maximum IR, to use as a measure of the degree of muscle IR and liver IR, respectively. Scaling was applied to facilitate result interpretation and to standardize IR measurements for both muscle and liver.

### Plasma Proteomics

In the DiOGenes cohort (14) we used the SOMAscan aptamer-based technology from the SomaLogic proteomics platform to measure a panel of 1128 plasma proteins. A continuous linear regression analysis was performed in R (version 4.3.1) to identify the plasma proteins that are associated with the degree of muscle or liver IR, utilizing the R-package “Limma” (17):


$$\begin{array}{rl} Y_{j}\;= & \beta_{0,j}+\beta_{1,j}\times IR_{muscle}+\beta_{2,j}\times IR_{liver}+\beta_{3,j}\times Sex\\ & +\beta_{4,j}\times Age+\beta_{5,j}\times Centre+\beta_{6,j}\times BMI+\varepsilon \end{array}$$

where *Y_j_* represents the protein abundance for each protein (j = 1 to 1128) in our panel, IR_muscle_ indicates the scaled degree of muscle IR (value between 0 and 1; see previous section), and IR_liver_ denotes the scaled degree of liver IR (value between 0 and 1; see previous section). In this linear regression analysis, we corrected for study center, sex, BMI, and age. Additionally, analyses on liver IR were adjusted for muscle IR and vice versa to assess the independent associations of muscle and liver IR with the protein abundance levels. Proteins were marked as differentially abundant when *P* < .05, with Benjamini-Hochberg false discovery rate correction. Next to this, also the log fold change has been calculated, where a negative fold change indicates a decreased abundance of the protein and a positive fold change indicates an increase in protein abundance in relation to the degree of muscle or liver IR.

### Pathway Overrepresentation Analysis

An overrepresentation analysis was performed on the differentially abundant proteins (*P* < .05; without correction for multiple testing), utilizing the “ClusterProfiler” package in R (version 4.8.2) (18, 19). To facilitate pathway analysis, SomaLogic identifiers for these proteins were converted to Entrez identifiers using the aptamer data from SomaLogic. However, this translation does not always result in a 1:1 mapping, as some proteins are encoded by multiple genes and vice versa, leading to a 1:N or N:1 mapping. Proteins with such mappings have been included in our pathway analysis but have been marked with bold-outlined circles in the visualizations.

Pathways were considered significantly enriched when (1) the adjusted *P*-value < .05 and (2) the pathway contained at least ∼5% of the total number of differentially abundant proteins. For the pathway analysis, the WikiPathways database has been consulted (20).

## Results

### Participant Characteristics

The proteomics subset of the DiOGenes cohort includes 535 relatively healthy adults with a mean age of 49.9 ± 0.3 years and a mean BMI of 34.4 ± 0.2 kg/m^2^. The study population consists of 61% of women. Participant characteristics can be found in Table 1.

**Table 1.** Clinical characteristics of the study participants

	Cohort (n = 535)	
	Mean ± SE	Range
Sex (males/females)	209/326	—
Age (years)	41.9 ± 0.3	24-63
BMI (kg/m^2^)	34.4 ± 0.2	25.6-52.0
Fasting glucose (mmol/L)	5.1 ± 0.03	1.8-9.4
Fasting insulin (mU/L)	11.9 ± 0.4	2.2-141
HOMA-IR (AU)	3.2 ± 0.1	0.6-37.1
Fasting FFAs (µmol/L)	641 ± 16	87-2614
Triglycerides (µmol/L)	1.4 ± 0.03	0.4-3.9
2 hours glucose (mmol/L)	6.7 ± 0.09	2.2-16.6
MISI (AU)	0.16 ± 0.006	0.001-1.1
HIRI (AU)	20.6 ± 0.3	7.4-58.6

The table includes mean values ± SEs.

Abbreviations: AU, arbitrary units; BMI, body mass index; FFA, free fatty acid; HIRI, hepatic insulin resistance index; HOMA-IR, homeostatic model assessment of insulin resistance; MISI, muscle insulin sensitivity index.

### Muscle and Liver IR Are Characterized by Distinct Plasma Protein Abundance Profiles

**Figure 1.**
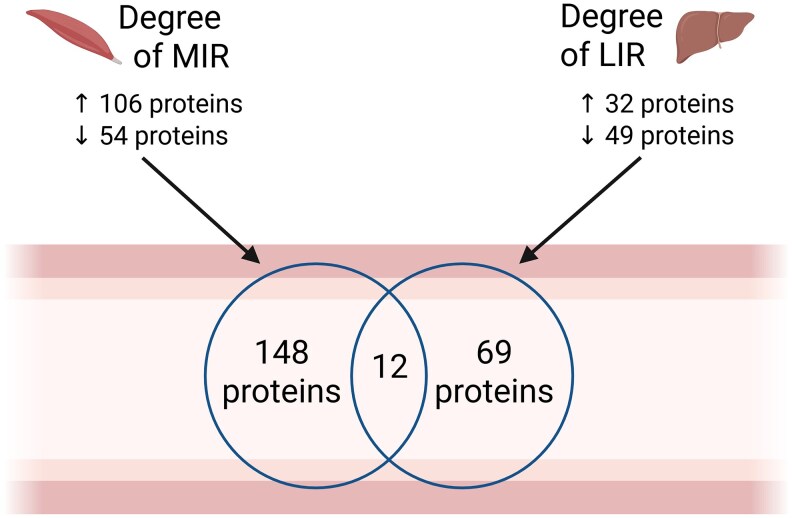
Venn diagrams displaying the number of differentially abundant proteins (*P* < .05) associated with muscle or liver insulin resistance. Figure created with BioRender.com.

To identify plasma proteins associated with muscle or liver IR, we performed continuous regression analysis. This analysis revealed that 160 proteins were independently associated with muscle IR, with 106 showing increased abundance and 54 showing decreased abundance as muscle IR increased [Fig. 1 and as Table S (21)]. In addition, 81 proteins were independently associated with liver IR, with 32 showing increased abundance and 49 showing decreased abundance with increasing liver IR [Fig. 1 and as Table S (21)]. Of these differentially abundant proteins, 12 were independently associated with both muscle and liver IR (Table 2).

**Table 2.** Proteins associated with both muscle and liver IR

			Muscle IR		Liver IR	
	Entrez ID	Protein name	LogFC	*P*-value	LogFC	*P*-value
1	Met	Hepatocyte growth factor receptor	−0.25	3.26E-02	−0.30	1.42E-02
2	BNP-32	Brain natriuretic peptide 32	0.62	3.92E-02	−0.66	3.31E-02
3	IGFBP-1	Insulin-like growth factor-binding protein 1	−0.72	3.80E-02	−1.94	7.66E-08
4	IGFBP-2	Insulin-like growth factor-binding protein 2	−0.64	8.38E-04	−1.07	8.95E-08
5	TSH	Thyroid-stimulating hormone	−0.71	1.85E-02	0.64	3.87E-02
6	IL-10	Interleukin-10	−0.84	6.65E-07	0.45	9.20E-03
7	cIAP-2	Baculoviral IAP repeat-containing protein 3	0.62	3.84E-02	−0.69	2.68E-02
8	NR1D1	Nuclear receptor subfamily 1 group D member 1	0.70	2.10E-03	−0.56	1.66E-02
9	IL-12 RB2	Interleukin-12 receptor subunit beta-2	0.64	4.94E-02	−0.85	1.19E-02
10	RET	Proto-oncogene tyrosine-protein kinase receptor Ret	0.68	7.69E-03	0.60	2.09E-02
11	CDON	Cell adhesion molecule-related/downregulated by oncogenes	−0.25	3.10E-02	0.30	1.38E-02
12	KI3S1	Killer cell immunoglobulin-like receptor 3DS1	0.79	1.50E-02	−0.72	3.06E-02

Of the differentially abundant proteins associated with either form of IR, 12 proteins were independently associated with both muscle and liver IR. The table includes the log fold changes and the unadjusted *P*-values, indicating the direction and magnitude of change.

Abbreviations: IR, insulin resistance; LogFC, log fold change.

After adjusting for multiple testing, 5 proteins were significantly associated with muscle IR and 5 with liver IR (q-value < 0.05) (Table 3). For muscle IR, these proteins include interleukin-10 , complement component C8, antithrombin II, kallikrein-11, and ectodysplasin-A. For liver IR, we identified a distinct set of significant proteins, including insulin-like growth factor-binding protein 1 (IGFBP-1) and 2 (IGFBP-2), adiponectin, C-C motif chemokine 23 and SHBG.

**Table 3.** Proteins significantly associated with muscle IR or liver IR after adjustment for multiple testing (adjusted *P*-value < .05)

	Name	Protein name	LogFC	Adjusted *P*-value
Muscle IR	IL-10	Interleukin-10	−0.84	7.50E-04
	Kallikrein 11	Kallikrein-11	−0.73	1.67E-02
	EDA	Ectodysplasin-A, secreted form	−0.67	1.67E-02
	C8	Complement component C8	−0.65	2.34E-02
	Antithrombin III	Antithrombin-III	−0.25	2.34E-02
Liver IR	IGFBP-1	Insulin-like growth factor-binding protein 1	−1.94	5.05E-05
	IGFBP-2	Insulin-like growth factor-binding protein 2	−1.07	5.05E-05
	Adiponectin	Adiponectin	−1.02	6.69E-04
	MPIF-1	C-C motif chemokine 23	−0.75	7.58E-04
	SHBG	Sex hormone-binding globulin	−1.22	2.16E-02

Abbreviations: LogFC, log fold change; IR, insulin resistance.

### Muscle IR Is Associated with Multiple Inflammation-related Pathways, While Liver IR Predominantly Associates with the Complement System Pathway

**Figure 2.**
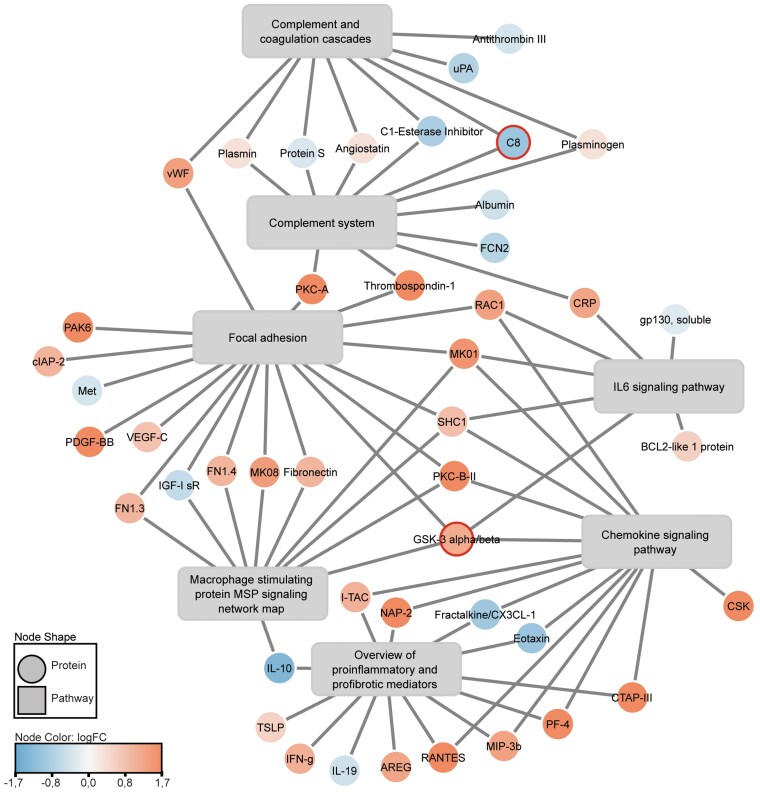
Network figure containing inflammatory pathways associated with muscle IR. Pathways are obtained from WikiPathways curated collection of human pathways. We only included relevant inflammatory pathways with *P* < .05 and that contain ≥ 5% of covered proteins (≥7 differentially abundant proteins). Each square represents a separate pathway. Plasma proteins are depicted as circles, in which red indicates a positive association with muscle IR and blue indicates a negative association with muscle IR. Proteins that do not have a 1:1 mapping from SomaLogic to Entrez ID have been marked with bold-outlined circles in the visualizations. Figure created with Cytoscape. Abbreviation: IR, insulin resistance.

To gain insights into the functional properties of the proteins that were significantly associated with muscle and liver IR, pathway enrichment analysis was applied. For the proteins that were associated with muscle IR, 51 pathways were found to be enriched (Table 4). Many of these pathways were related to inflammation, indicating that muscle IR is characterized by a proinflammatory profile. This includes pathways involved in focal adhesion, proinflammatory mediators, chemokine signaling, interleukin 6 (IL-6) signaling, and the complement system (Fig. 2). Additionally, pathway analysis revealed an enrichment of the PI3K-Akt signaling pathway.

**Table 4.** Significantly enriched pathways associated with muscle IR

Pathway description	GeneRatio	Adjusted *P*-value
Pleural mesothelioma	27/143	1.76E-06
Focal adhesion	16/143	5.12E-05
Network map of SARS-CoV-2 signaling pathway	16/143	7.77E-05
VEGFA VEGFR2 signaling	23/143	7.77E-05
Wnt signaling pathway	8/143	7.77E-05
EGFR tyrosine kinase inhibitor resistance	10/143	8.94E-05
Overview of proinflammatory and profibrotic mediators	12/143	9.82E-05
Kit receptor signaling pathway	8/143	1.21E-04
Wnt signaling	11/143	1.37E-04
Prolactin signaling pathway	9/143	1.50E-04
Chemokine signaling pathway	13/143	1.54E-04
Cell cycle	11/143	1.79E-04
IL6 signaling pathway	7/143	1.94E-04
Ras signaling	13/143	3.54E-04
AGE RAGE pathway	8/143	3.54E-04
TGF β signaling pathway	11/143	3.54E-04
Sudden infant death syndrome susceptibility pathways	12/143	3.69E-04
PI3K Akt signaling pathway	18/143	3.70E-04
Urotensin II-mediated signaling pathway	8/143	3.97E-04
ErbB signaling pathway	9/143	3.97E-04
B-cell receptor signaling pathway	9/143	4.55E-04
Complement system	9/143	5.66E-04
Oncostatin M signaling pathway	7/143	6.64E-04
Complement and coagulation cascades	7/143	7.60E-04
MicroRNAs in cardiomyocyte hypertrophy	9/143	9.02E-04
Myometrial relaxation and contraction pathways	11/143	9.02E-04
Macrophage-stimulating protein signaling network map	8/143	9.43E-04
Complement system in neuronal development and plasticity	9/143	1.04E-03
Acute viral myocarditis	8/143	1.25E-03
Orexin receptor pathway	8/143	1.40E-03
Gastrin signaling pathway	9/143	1.59E-03
Hippo signaling regulation pathways	8/143	2.56E-03
MAPK signaling pathway	13/143	2.88E-03
Regulatory circuits of the STAT3 signaling pathway	7/143	3.23E-03
Focal adhesion PI3K Akt mTOR signaling pathway	14/143	4.99E-03
Neuroinflammation and glutamatergic signaling	9/143	5.03E-03
T-cell activation SARS-CoV-2	7/143	5.49E-03
Allograft rejection	7/143	6.08E-03
Spinal cord injury	8/143	6.77E-03
CRH signaling pathway	7/143	6.80E-03
Calcium regulation in cardiac cells	9/143	7.24E-03
Insulin signaling	9/143	8.95E-03
CKAP4 signaling pathway map	7/143	1.40E-02
HDAC6 interactions in the central nervous system	7/143	1.44E-02
Osteoblast differentiation and related diseases	7/143	1.95E-02
Hepatitis B infection	8/143	1.95E-02
Epithelial to mesenchymal transition in colorectal cancer	8/143	2.75E-02
Brain-derived neurotrophic factor signaling pathway	7/143	3.44E-02
Regulation of actin cytoskeleton	7/143	4.92E-02
Alzheimer’s disease	10/143	4.92E-02
Vitamin D receptor pathway	8/143	4.95E-02

Abbreviation: IR, insulin resistance.

**Figure 3.**
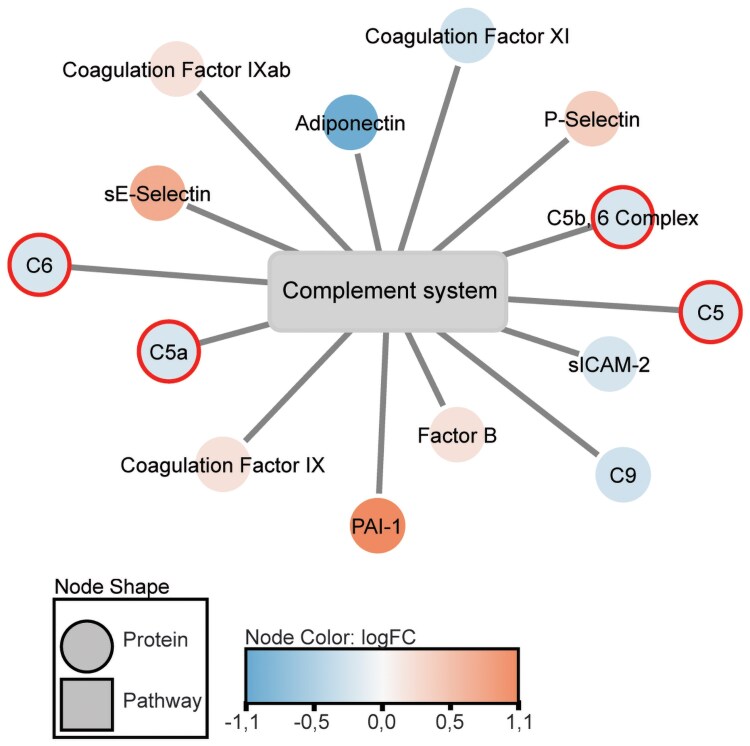
Network figure visualizing complement system pathway associated with liver IR. We combined 3 complement-related pathways with a *P* < .05 and that contain ≥ 5% of covered proteins (≥4 differentially abundant proteins), namely “complement activation,” “complement and coagulation cascades,” and “complement system.” Plasma proteins are depicted as circles, in which red indicates a positive association with the degree of liver IR and blue indicates a negative association with the degree of liver IR. Proteins that do not have a 1:1 mapping from SomaLogic to Entrez ID have been marked with bold-outlined circles in the visualizations. Figure created with Cytoscape. Abbreviation: IR, insulin resistance.

Notably, in contrast with muscle IR, only 11 pathways were found to be associated with liver IR (Table 5), and these pathways were primarily related to the complement system. For clarity, closely related complement system pathways from the WikiPathways database were combined in the visualization (Fig. 3). These include “complement activation,” “complement and coagulation cascades,” and “complement system.”

**Table 5.** Significantly enriched pathways associated with liver IR

Pathway description	GeneRatio	Adjusted *P*-value
Complement-mediated inflammation of pulmonary alveolus in COVID-19 hypothetical pathway	4/61	1.48E-04
Complement activation	4/61	1.49E-03
T-cell modulation in pancreatic cancer	4/61	1.02E-02
Complement and coagulation cascades	5/61	2.40E-03
Orexin receptor pathway	5/61	1.16E-02
Complement system in neuronal development and plasticity	5/61	2.13E-02
Adipogenesis	5/61	3.76E-02
Allograft rejection	6/61	2.18E-03
Vitamin D receptor pathway	6/61	3.75E-02
Complement system	9/61	5.37E-06
Network map of SARS-CoV-2 signaling pathway	9/61	1.49E-03

Abbreviation: IR, insulin resistance.

The overlapping pathways associated with both muscle and liver IR are mainly associated to the complement system, such as “complement and coagulation cascades,” “complement system in neuronal development and plasticity,” and “complement system.” Additionally, other shared pathways include the “orexin receptor pathway,” “allograft rejection,” “vitamin D receptor pathway,” and “network map of SARS-CoV-2 signaling pathway.” Notably, despite the overlap in enrichment of the complement pathways, the specific proteins driving the enrichment of these pathways differ between muscle and liver IR, with no overlap between the protein sets (data not shown).

## Discussion

Individuals with pronounced muscle IR or pronounced liver IR have distinct metabolic traits, suggesting variability in cardiometabolic disease development (22, 23). The aim of the current study was to investigate which proteins are independently associated with liver or muscle IR and in which pathways these proteins are involved. We performed a continuous regression analysis on the DiOGenes cohort, including 535 individuals with available plasma proteome data. Our study found 160 proteins to be specifically associated with muscle IR and 81 proteins with liver IR, with 12 proteins that were independently associated with both. Pathway enrichment analysis further revealed that muscle IR was associated with multiple inflammation-related pathways, including IL-6 signaling, focal adhesion, and the complement system, while liver IR was predominantly associated with the complement system.

One of the pathways associated with muscle IR was the inflammation-related IL-6 signaling pathway, which has previously been linked to insulin signaling and glucose homeostasis (24). Remarkably, despite this enrichment, we did not observe significant changes in the abundance of IL-6, and instead, the pathway was driven by different proteins, including CRP and GP130 (also known as IL-6 receptor unit β). Also, other inflammation-related proteins were found to be differentially abundant. Interleukin 10 and antithrombin III, both with anti-inflammatory properties (25, 26), were downregulated with increasing muscle IR. These findings align with previous evidence of a proinflammatory adipose tissue and systemic profile associated with muscle IR (8). Specifically, van der Kolk et al showed that individuals with muscle IR have both an upregulation of inflammatory genes in the abdominal subcutaneous adipose tissue and an increased systemic inflammatory profile, and the association of muscle IR, but not liver IR, with systemic low-grade inflammation was established using 2 independent cohorts (8). Interestingly, our analysis also shows that the focal adhesion pathway was enriched in relation to muscle IR. This pathway plays an important role in cellular signaling, particularly in mediating interactions between cells and the extracellular matrix (27).

Several pathways related to the complement system were enriched for muscle IR as well as for liver IR. The complement system, a vital component of the human innate immune system, consists of proteins primarily synthesized in the liver (28) and has been linked to metabolic diseases, mainly via complement component C3 and C5 (29). Notably, C5 was significantly downregulated for liver IR in our study. C5 has previously been identified as a proinflammatory mediator, and decreased levels have been associated with a decreased ability of the liver to repair itself (30). Interestingly, while several complement system-related pathways were found to be enriched in both muscle and liver IR, the proteins driving these pathways were distinct. This indicates that the underlying molecular drivers may differ, highlighting the tissue-specific nature of muscle and liver IR. The differential proteome profiles associated with muscle and liver IR are in line with previous studies from our lab, which showed distinct molecular and metabolic profiles in individuals with muscle and liver IR, including variations in serum metabolome profiles (6), plasma lipidome (7), and subcutaneous adipose tissue transcriptome profiles (8). These tissue-specific IR phenotypes have also been shown to respond differentially to dietary macronutrient composition, with considerable improvements in cardiometabolic health when diets were tailored accordingly (9).

The differentially abundant proteins that are associated with liver IR include IGFBP-1 and IGFBP-2, proteins that are primarily expressed in the liver. Our results indicated a downregulation of IGFBP-1 and IGFBP-2, and low levels of these proteins are linked to IR and T2D (31, 32). A decrease of IGFBP-2 is also associated with an increased risk of metabolic dysfunction-associated steatotic liver disease (33) and is linked to an increase in plasma triglycerides (32). Of note, increased postprandial triglyceride levels were previously found in individuals with pronounced liver IR compared to individuals with more pronounced muscle IR (34). Interestingly, our results also showed a downregulation of SHBG associated with liver IR. SHBG is predominantly synthesized in the liver, and a decreased abundance is linked with the development of metabolic dysfunction-associated steatotic liver disease and associated with an increased risk of IR and T2D (35, 36). Furthermore, we observed a decreased abundance of adiponectin in relation to liver IR. Adiponectin is predominantly secreted by adipose tissue and plays an important role in glucose metabolism. In an in vitro study, it was demonstrated that adiponectin significantly enhances hepatic insulin sensitivity by lowering hepatic glucose production (37). We hypothesize that the close connection between visceral adipose tissue and the liver via the portal vein may explain the downregulation of adiponectin observed with increased liver IR.

One of the strengths of our study is the use of a large dataset, which includes measurements from a comprehensive proteomics panel covering 1128 proteins in 535 individuals. By employing a continuous regression model rather than a group-based approach, we were able to include data from all participants and accounted for the fact that individuals often exhibit a combination of muscle and liver IR rather than falling into discrete categories. This approach increases statistical power and helps uncover independent mechanisms of muscle and liver IR, but it also means that the identified proteins do not serve as direct biomarkers. A limitation of our study is the conversion of SomaLogic identifiers to the more widely used Entrez identifiers. While this step is essential for functional pathway analysis, it inevitably leads to a loss of information on posttranslational modifications. Additionally, the degree of muscle and liver IR has been estimated by a 5-point OGTT rather than the gold-standard method, the hyperinsulinemic-euglycemic clamp. Nevertheless, the OGTT has previously been validated against this gold-standard method (15), and it may actually provide a better reflection of human physiology since it also includes the influence of the gastrointestinal tract.

In the current study, plasma proteomics data were collected from participants in a fasting state, whereas both muscle and hepatic IR indexes were derived from postprandial responses following an oral glucose load (15). It would therefore be of interest to study plasma protein profiles after ingestion of a meal (38). In support of this, recent findings indicated that individuals with pronounced liver IR exhibit more adverse postprandial metabolite responses compared to individuals with muscle IR (34). Based on these insights, we would propose a follow-up study incorporating proteomics measurements at multiple time points during an OGTT or high-fat mixed meal. Such an approach would enable a detailed exploration of postprandial protein dynamics, particularly in relation to tissue-specific IR. This will yield additional insights into the underlying mechanisms of the liver IR and muscle IR phenotypes and their differential responses to the same intervention. Combining this extensive proteomic profiling with previously acquired knowledge will offer a more integrative view on the distinct characteristics of these phenotypes. This contributes to a more elaborate mechanistic understanding of their differential response to dietary interventions, representing an important step toward the development of more targeted, group-based interventions to alleviate tissue-specific IR.

In conclusion, our results show that IR in muscle and liver is linked to distinct plasma protein profiles. Muscle IR is characterized by a pronounced inflammatory profile, whereas liver IR is associated with changes in the complement system. The differences in circulating proteome profiles further highlight the heterogeneity in the development of cardiometabolic diseases. This understanding supports the development of more targeted, tissue-specific interventions, such as personalized nutrition, to prevent the progression to more severe cardiometabolic diseases as underlying differences between these metabolic profiles are increasingly elucidated.

## Data Availability

The data presented in this manuscript are available from the corresponding author on reasonable request.

## Clinical trial registration

https://ClinicalTrials.gov, identifier: NCT00390637.
